# Digital dissection of the masticatory muscles of the naked mole-rat, *Heterocephalus glaber* (Mammalia, Rodentia)

**DOI:** 10.7717/peerj.448

**Published:** 2014-06-17

**Authors:** Philip G. Cox, Chris G. Faulkes

**Affiliations:** 1 Hull York Medical School, University of Hull, Hull, UK; 2 School of Biological and Chemical Sciences, Queen Mary University of London, UK

**Keywords:** Naked mole-rat, Masticatory muscles, MicroCT, Virtual reconstruction, Bathyergidae, Rodents

## Abstract

The naked mole-rat, *Heterocephalus glaber*, of the family Bathyergidae is a subterranean rodent that feeds on underground roots and tubers and digs extensive tunnel systems with its incisors. It is a highly unusual mammal with regard to its social structure, longevity, pain insensitivity and cancer resistance, all of which have made it the subject of a great deal of research in recent years. Yet, much of the basic anatomy of this species remains undocumented. In this paper, we describe the morphology of the jaw-closing musculature of the naked mole-rat, as revealed by contrast-enhanced micro-computed tomography. This technique uses an iodine stain to enable the imaging of soft tissues with microCT. The iodine-enhanced scans were used to create 3D reconstructions of the naked mole-rat masticatory muscles from which muscle masses were calculated. The jaw-closing musculature of *Heterocephalus glaber* is relatively very large compared to other rodents and is dominated by the superficial masseter, the deep masseter and the temporalis. The temporalis in particular is large for a rodent, covering the entirety of the braincase and much of the rear part of the orbit. The morphology of the masseter complex described here differs from two other published descriptions of bathyergid masticatory muscles, but is more similar to the arrangement seen in other rodent families. The zygomaticomandibularis (ZM) muscle does not protrude through the infraorbital foramen on to the rostrum and thus the naked mole-rat should be considered protrogomorphous rather than hystricomorphous, and the morphology is consistent with secondarily lost hystricomorphy as has been previously suggested for Bathyergidae. Overall, the morphology of the masticatory musculature indicates a species with a high bite force and a wide gape–both important adaptations for a life dominated by digging with the incisors.

## Introduction

The naked mole-rat, *Heterocephalus glaber* (Rüppell, 1842), is found in the hot, dry regions of the Horn of Africa (Honeycutt et al., 1991), and is probably the most well-known member of the Bathyergidae — a highly specialised group of subterranean rodents known as the African mole-rats or blesmols. Like all bathyergids except *Bathyergus* (which mainly uses its foreclaws), the naked mole-rat digs with its incisors and is able to close its lip folds behind the incisors to prevent soil from entering the mouth during tunnelling (Tucker, 1981). Naked mole-rats feed exclusively on underground roots and tubers, and their ‘chisel-toothed’ digging through hard, compact soil in the search for widely dispersed food resources is likely to have influenced the musculature of the jaw. The naked mole-rat is the only species within the genus *Heterocephalus*, and phylogenetic analyses indicate that it is the most basal extant species of the family (Allard & Honeycutt, 1992), with an estimated divergence from the other bathyergid genera between 40 and 48 million years ago (Faulkes et al., 2004), or 33–35 million years ago, depending on the fossil calibration of the molecular clock (Faulkes et al., 2011; Ingram, Burda & Honeycutt, 2004). On this basis, some authors place the naked mole-rat in its own subfamily, the Heterocephalinae, distinct from the remaining genera in the Bathyerginae (e.g., Wilson & Reeder, 2005). The naked mole-rat first came to prominence scientifically when it was shown that its complex social structure is in fact a rare example of eusociality in vertebrates (Jarvis, 1981; Jarvis, 1991). That is, naked mole-rats have a caste system analogous to that of ants and termites, with a breeding female, or queen, at the top of the hierarchy, and smaller, non-breeding workers at the bottom. Since then, naked mole-rats have been discovered to possess many other unusual qualities that have placed them in the forefront of research in a number of fields. For example, naked mole-rats appear to be insensitive to acid-induced pain (Smith et al., 2011), extraordinarily resistant to cancer (Seluanov et al., 2009), and extremely long-lived for a small mammal (Buffenstein, 2005).

According to recent estimates (Faulkes & Bennett, 2013), the Bathyergidae comprises thirty or more species, in six genera, and is inferred to have originated in the Eocene of Africa (Huchon & Douzery, 2001) although the earliest known fossils are Miocene in age (Faulkes et al., 2004; Mein & Pickford, 2008). On the basis of lower jaw and inner ear morphology, the bathyergids were considered members of the Hystricognathi by Tullberg (1899). Subsequent works tended to preserve this relationship, with the Bathyergidae frequently being closely allied to two other families of Old World hystricognaths, the Thryonomyidae and Petromuridae (Landry, 1957; Wood, 1974). However, a degree of doubt remained over the placement of bathyergids owing to the unusual morphology of their masticatory muscles. Almost all living rodents can be classified as sciuromorph (squirrel-like), myomorph (mouse-like) or hystricomorph (porcupine-like) based on the morphology of the masseter muscle (Brandt, 1855; Wood, 1965). Most members of the Hystricognathi are defined as being hystricomorph, owing to the possession of an enlarged infraorbital foramen, through which a substantial portion of the zygomaticomandibularis (ZM) muscle (or medial masseter) extends to take an origin on the rostrum. However, in most Bathyergidae, the infraorbital foramen is small, simply transmitting the infraorbital artery and the infraorbital branch of the maxillary nerve (Maier & Schrenk, 1987). Thus, no part of the masticatory musculature attaches to the rostrum. This morphology is termed protrogomorphous, and is thought to be the ancestral condition for rodents also seen in many Eocene fossil taxa (Wood, 1965) and the extant mountain beaver, *Aplodontia rufa* (Druzinsky, 2010), although claims of hystricomorphy in *Aplodontia* have also been made (Eastman, 1982). It should be noted, however, that a moderate enlargement of the infraorbital foramen is seen in two recently-split extant genera of blesmols, *Cryptomys* (Boller, 1970; Morlok, 1983) and *Fukomys* (Van Daele, Herrel & Adriaens, 2009), as well as in fossil genera from the Miocene of East Africa, (Lavocat, 1973; Lavocat, 1974). In *Cryptomys* and *Fukomys* this enlargement is accompanied by a very limited extension of the ZM through the foramen on to the rostrum (Boller, 1970; Van Daele, Herrel & Adriaens, 2009).

Given the variable morphology of the ZM and infraorbital foramen seen amongst extant and fossil genera, bathyergid protrogomorphy was the subject of some debate for a number of years, particularly with regard to its evolutionary history: Does the bathyergid condition represent retention of the primitive condition or is the morphology secondarily derived? Tullberg (1899), Wood (1965) and Wood (1985) believed that bathyergids show the ancestral condition, and that *Cryptomys* demonstrates nascent hystricomorphy. On the other hand, Landry (1957), Lavocat (1974) and Luckett & Hartenberger (1985) were of the opinion that bathyergids evolved from a hystricomorph ancestor, and that their current morphology represents a reversal to the primitive condition. Maier & Schrenk (1987) added support to the latter view by showing that small bundles of fibres of the ZM muscle protrude through the infraorbital foramen early in development in two genera of blesmols, *Bathyergus* and *Georychus*, but subsequently retreat from the rostrum, and are absent at birth. More recently, molecular analyses have supported a close evolutionary association of the Bathyergidae, Thryonomyidae (cane-rats) and Petromuridae (dassie rat), within the Hystricognathi (Blanga-Kanfi et al., 2009; Fabre et al., 2012). Given the clear hystricomorphy of *Thryonomys* and *Petromus* (Woods, 1972), under this phylogenetic arrangement it would be more parsimonious to assume that blesmols evolved from a hystricomorph ancestor, than that they retained the protrogomorph condition, and hence that hystricomorphy evolved at least three times independently within the hystricognath radiation (in Hystricidae; in the ancestor of Thryonomyidae + Petromuridae; and in the Caviomorpha), as well as once in the Ctenodactyloidea, the sister-clade to Hystricognathi.

Despite the controversy surrounding mole-rat muscular morphology, there are very few detailed descriptions of the masticatory musculature of bathyergids. In his monumental study of rodent anatomy, Tullberg (1899) included three bathyergid species: *Georychus capensis*, *Georychus coecutiens* (now synonymised with *Cryptomys hottentotus* according to Wilson & Reeder (2005)) and *Bathyergus maritimus* (now *B. suillus*). As mentioned above, the jaw-closing muscles of *Cryptomys hottentotus* were also described by Boller (1970). Morlok (1983) examined all five genera of bathyergids recognised at the time (*Bathyergus*, *Cryptomys*, *Georychus*, *Heliophobius* and *Heterocephalus*), but only provided detailed descriptions of the musculature of *Cryptomys*. More recently, the jaw-closing muscles of the genus *Fukomys* (formerly part of *Cryptomys* Kock et al., 2006) were briefly described by Van Daele, Herrel & Adriaens (2009). The Bathyergidae were excluded from the comparative study of New and Old World hystricomorphs of Woods (1972) owing to their perceived protrogomorphy. This study aims to fill at least part of this gap in the comparative literature by examining the masticatory musculature of one particularly notable absentee, the naked mole-rat.

One reason underlying the lack of published literature on the masticatory musculature of the naked mole-rat may be the small size of this species, which renders traditional dissection more difficult than for larger species. However, its small size also makes the naked mole-rat an ideal candidate for digital dissection via contrast-enhanced micro-computed tomography (microCT) and virtual reconstruction. This technique is based on the work of Metscher (2009) and Jeffery et al. (2011) and involves the staining of biological specimens with iodine potassium iodide (I_2_KI) to enable the visualisation of soft tissues with microCT. The musculature of several rodent species has already been successfully imaged using this technique (Cox & Jeffery, 2011; Hautier, Lebrun & Cox, 2012; Baverstock, Jeffery & Cobb, 2013) as well as that of other vertebrates (Tsai & Holliday, 2011; Düring et al., 2013; George & Holliday, 2013; Gignac & Kley, 2014; Lautenschlager, Bright & Rayfield, 2014). The aim of this study is to provide a description of the jaw-closing musculature of *H. glaber*. Given the demands made on the masticatory system by the chisel-tooth digging by which it excavates tunnels, knowledge of the masticatory muscles is of prime importance for understanding the biology of the naked mole-rat. The descriptions provided here will enable comparisons to be made between the naked mole-rat and the other genera of bathyergids already described in the literature, as well as with other members of the Hystricognathi, both fossorial and non-fossorial.

## Materials and Methods

### Sample and scanning

Three naked mole-rat individuals were obtained from collections held at Queen Mary University of London. The specimens were all non-breeding workers and had been preserved in 95% ethanol for several years. In order to visualise the bony morphology, the specimens were imaged using microCT at the Department of Engineering, University of Hull. The scans were performed at 100 kV and 37 µA, with a copper filter averaging two frames per projection. Isometric voxel sizes ranged between 0.015 and 0.02 mm. Following the initial scanning, the specimens were immersed in a 5% solution of I_2_KI dissolved in phosphate-buffered formal saline for a period of two weeks. The stained specimens were then microCT scanned, again at the Department of Engineering, University of Hull. The scan was performed at 80 kV and 60 µA without a filter, with 4000 projections averaging 2 frames per projection, and using a beryllium target. Voxels were isometric and ranged between 0.022 and 0.024 mm in size.

### Digital reconstruction

Only one of the contrast-enhanced microCT scans (corresponding to specimen ‘Hetero3’) was sufficiently well-resolved to enable virtual reconstruction of the masticatory muscles. This scan was imported as a stacked TIFF into Avizo 8.0 (FEI Visualization Sciences Group, Burlington, MA, USA), and the masticatory muscles of the right side of the head were reconstructed. Although the muscles were clearly visible as individual components, the contrast difference between muscle and bone was not sufficiently different to enable automatic thresholding of the muscles. Thus manual segmentation was employed to produce the 3D muscle reconstructions. The cranium and mandible were also reconstructed to facilitate the visualisation of attachment areas. However, the bony components were reconstructed from the initial unstained scans to allow automatic thresholding. Bone and muscle reconstructions were then brought together and aligned in Avizo 8.0 to produce high resolution figures and movies. Downsampled surface files of the skull, mandible and muscles were combined using Adobe 3D Reviewer (Adobe Systems Inc., San Jose, CA, USA) to produce a 3D interactive PDF (File S1) following the method outlined by Lautenschlager (2014). The reconstructed surface file, along with the microCT scan, was deposited in Hydra, the University of Hull data repository, under the accession number 8475 (https://hydra.hull.ac.uk/resources/hull:8475). The remaining two scans, whilst not of sufficient quality for digital reconstruction, were at least detailed enough to provide comparisons to Hetero3, and were of help in determining muscle morphology.

### Analysis

Muscle volumes were calculated by Avizo 8.0 and converted to masses assuming muscle density of 1.0564 g/cm^3^ (Murphy & Beardsley, 1974). It was clear from the microCT images that the specimen had suffered extensive soft tissue shrinkage. This is likely to be a result of the iodine staining (Vickerton, Jarvis & Jeffery, 2013) as well as the lengthy preservation time. Therefore the absolute mass of each masticatory muscle should be approached with some caution. Vickerton, Jarvis & Jeffery (2013) provided estimates of percentage shrinkage for various concentrations of I_2_KI and staining durations, but calculation of degree of muscle shrinkage is not possible in this study as it is unknown exactly how long the specimens had been in ethanol before staining. However, the muscle attachment sites, their relative positions and their relationship to other anatomical structures were not affected by shrinkage. Moreover, the preservation and staining techniques affect all muscles equally, so the topology and relative proportions of the muscles can be analysed with confidence. Condylobasal cranial length (the midline distance along the cranial base from the anterior extremity of the premaxillae to the margin of the foramen magnum) of the specimen was measured to be 18.9 mm.

The arrangement of jaw-closing muscles in the naked mole-rat revealed by contrast-enhanced microCT was compared to previously published descriptions of masticatory muscles in other bathyergids: Tullberg (1899), Boller (1970), Morlok (1983) and Van Daele, Herrel & Adriaens (2009). In addition, to understand how naked mole-rat masticatory muscles are similar to or differ from rodents more generally, the results here were compared to published descriptions of other rodents including sciuromorphs, myomorphs and hystricomorphs, as well as the only living protrogomorph, *Aplodontia rufa*. The literature consulted was as follows: Müller, 1933 (*Hydrochoerus*); Greene, 1935 (*Rattus*); Schumacher & Rehmer, 1962 (*Cavia*, *Rattus*); Wood, 1965 (*Marmota*, *Myocastor*, *Ondatra*); Turnbull, 1970 (*Sciurus*, *Rattus*, *Hystrix*); Woods, 1972 (*Proechimys*, *Echimys*, *Isothrix*, *Mesomys*, *Myocastor*, *Octodon*, *Ctenomys*, *Erethizon*, *Cavia*, *Chinchilla*, *Dasyprocta*, *Thryonomys*, *Petromus*); Weijs, 1973 (*Rattus*); Woods & Howland, 1979 (*Capromys*, *Geocapromys*, *Plagiodontia*, *Myocastor*); Woods & Hermanson, 1985 (*Capromys*, *Geocapromys*, *Plagiodontia*, *Myocastor*, *Echimys*, *Octodon*, *Erethizon*, *Coendou*, *Dasyprocta*, *Atherurus*, *Thryonomys*, *Petromus*); Offermans & De Vree, 1989 (*Pedetes*); Ball & Roth, 1995 (*Sciurus*, *Microsciurus*, *Sciurillus*, *Tamiasciurus*, *Tamias*, *Glaucomys*); Thorington & Darrow, 1996 (*Aplodontia*, *Paraxerus*, *Funisciurus*, *Myosciurus*, *Heliosciurus*, *Protoxerus*, *Funambulus*, *Calliosciurus*, *Tamiops*, *Xerus*, *Atlantoxerus*, *Ratufa*); Olivares, Verzi & Vassallo, 2004 (*Aconaemys*, *Octomys*, *Tympanoctomys*, *Spalacopus*, *Octodon*, *Octodontomys*); Satoh & Iwaku, 2004 (*Cricetulus*, *Mesocricetus*, *Phodopus*, *Tscherkia*); Satoh & Iwaku, 2006 (*Onychomys*); Satoh & Iwaku, 2009 (*Neotoma*, *Peromyscus*); Druzinsky, 2010 (*Aplondontia*, *Cynomys*, *Tamias*, *Marmota*, *Ratufa*, *Sciurus*, *Thomomys*); Hautier & Saksiri, 2009 (*Laonastes*); Hautier, 2010 (*Ctenodactylus*); Cox & Jeffery, 2011 (*Cavia*, *Rattus*, *Sciurus*); Baverstock, Jeffery & Cobb, 2013 (*Mus*); Becerra et al., 2014 (*Chinchilla*, *Ctenomys*, *Octodon*).

## Results

**Figure 1:**
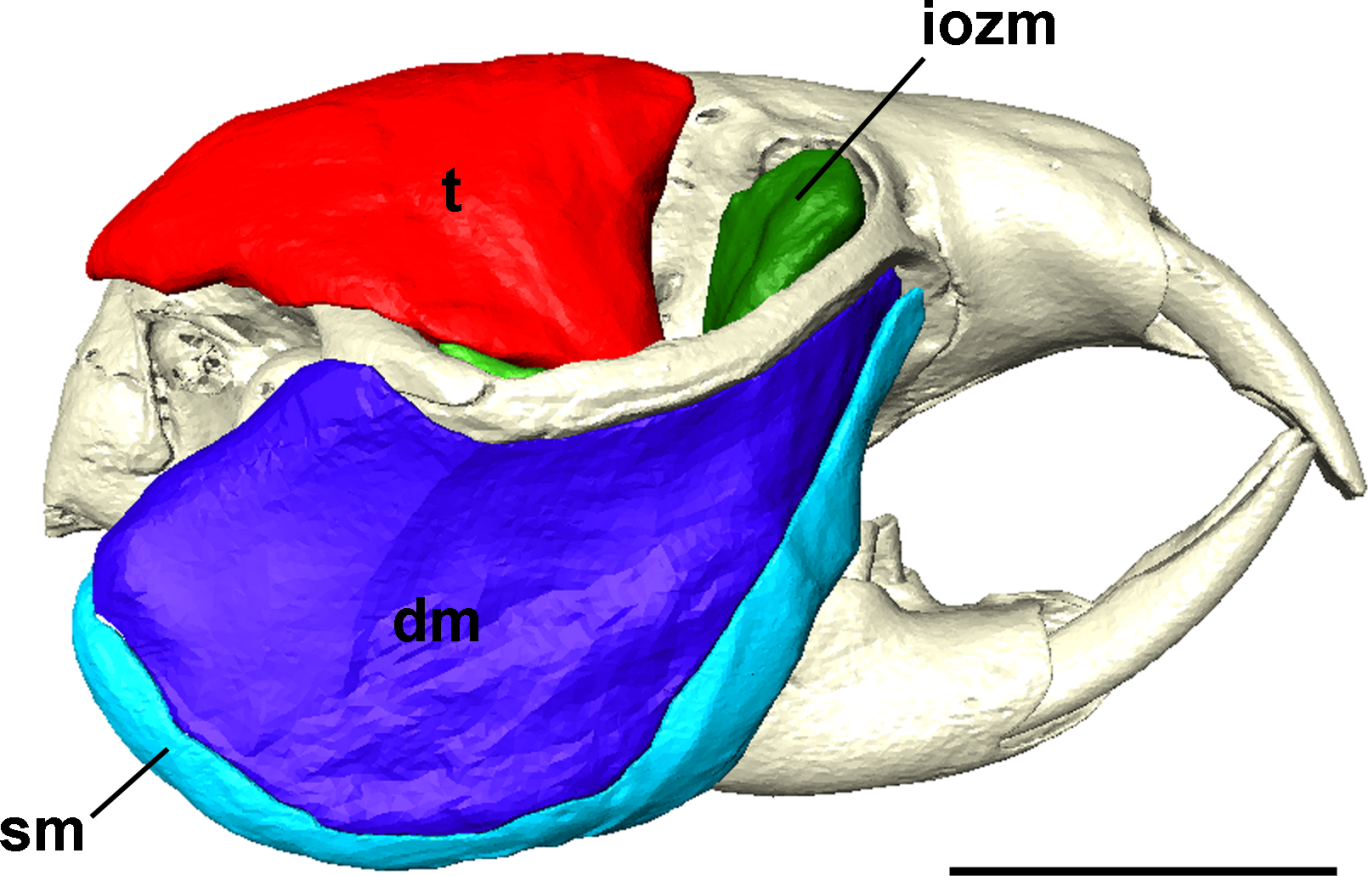
Masticatory muscles of *Heterocephalus glaber*. Right lateral view of a 3D reconstruction of the cranium, mandible and masticatory muscles of *Heterocephalus glaber*. Abbreviations: iozm, infraorbital zygomaticomandibularis; dm, deep masseter; sm, superficial masseter; *t*, temporalis. Scale bar = 5 mm.

**Figure 2:**
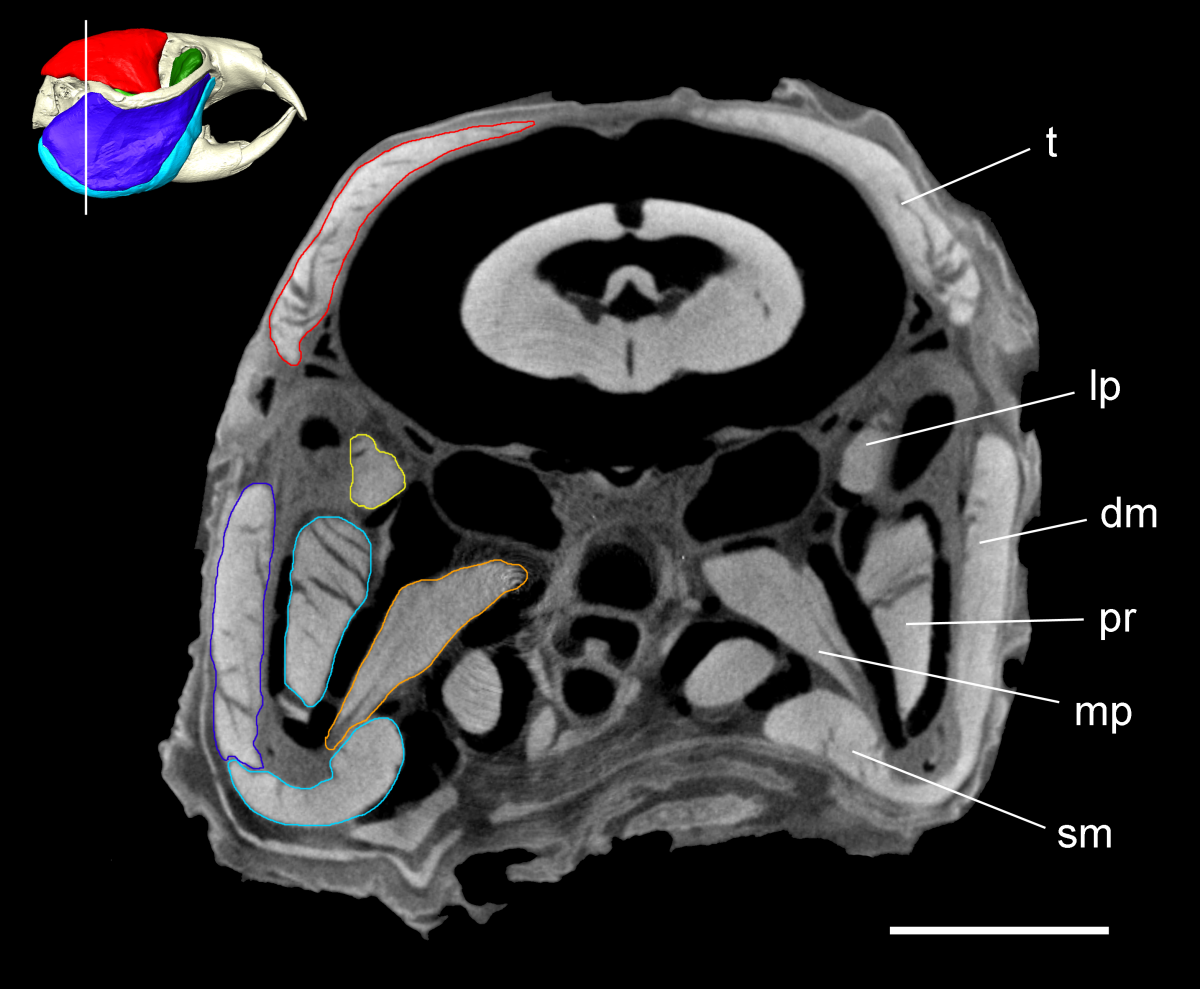
Coronal microCT slice of *Heterocephalus glaber*. Coronal microCT slice through the head of *Heterocephalus glaber* stained with iodine potassium iodide. Abbreviations: dm, deep masseter (dark blue); lp, lateral pterygoid (yellow); mp, medial pterygoid (orange); pr, *pars reflexa* of the superficial masseter (light blue); sm, superficial masseter (light blue); *t*, temporalis (red). White line on 3D reconstruction shows position of slice. Scale bar = 5 mm.

**Figure 3:**
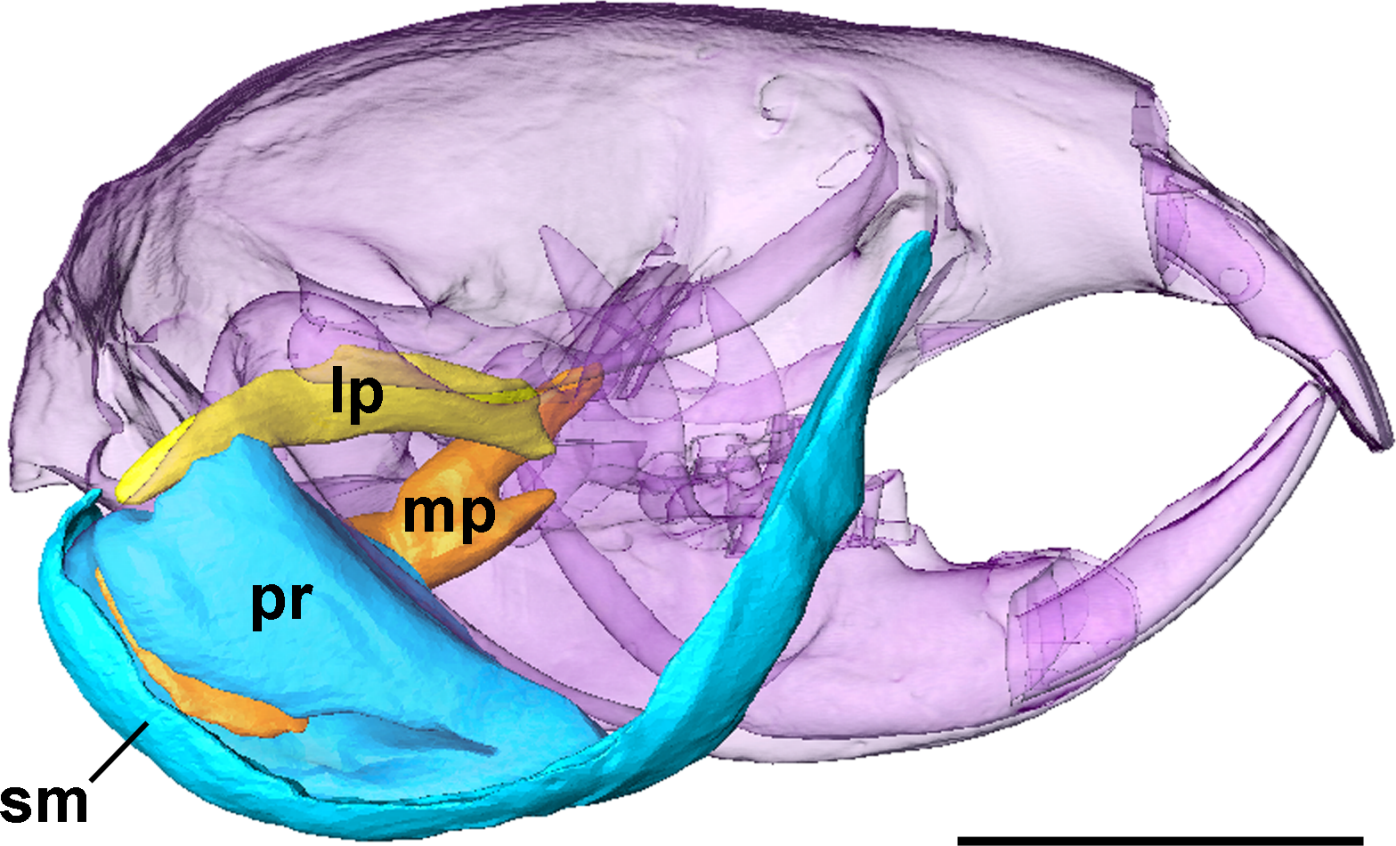
Superficial masseter and pterygoid muscles of *Heterocephalus glaber*. Right lateral view of a 3D reconstruction of the cranium, mandible, superficial masseter and pterygoid muscles of Heterocephalus glaber. Cranium and mandible transparent for visualisation of muscles attaching to medial mandibular surface. Abbreviations: lp, lateral pterygoid; mp, medial pterygoid; pr, *pars reflexa* of the superficial masseter; sm, superficial masseter. Scale bar = 5 mm.

**Figure 4:**
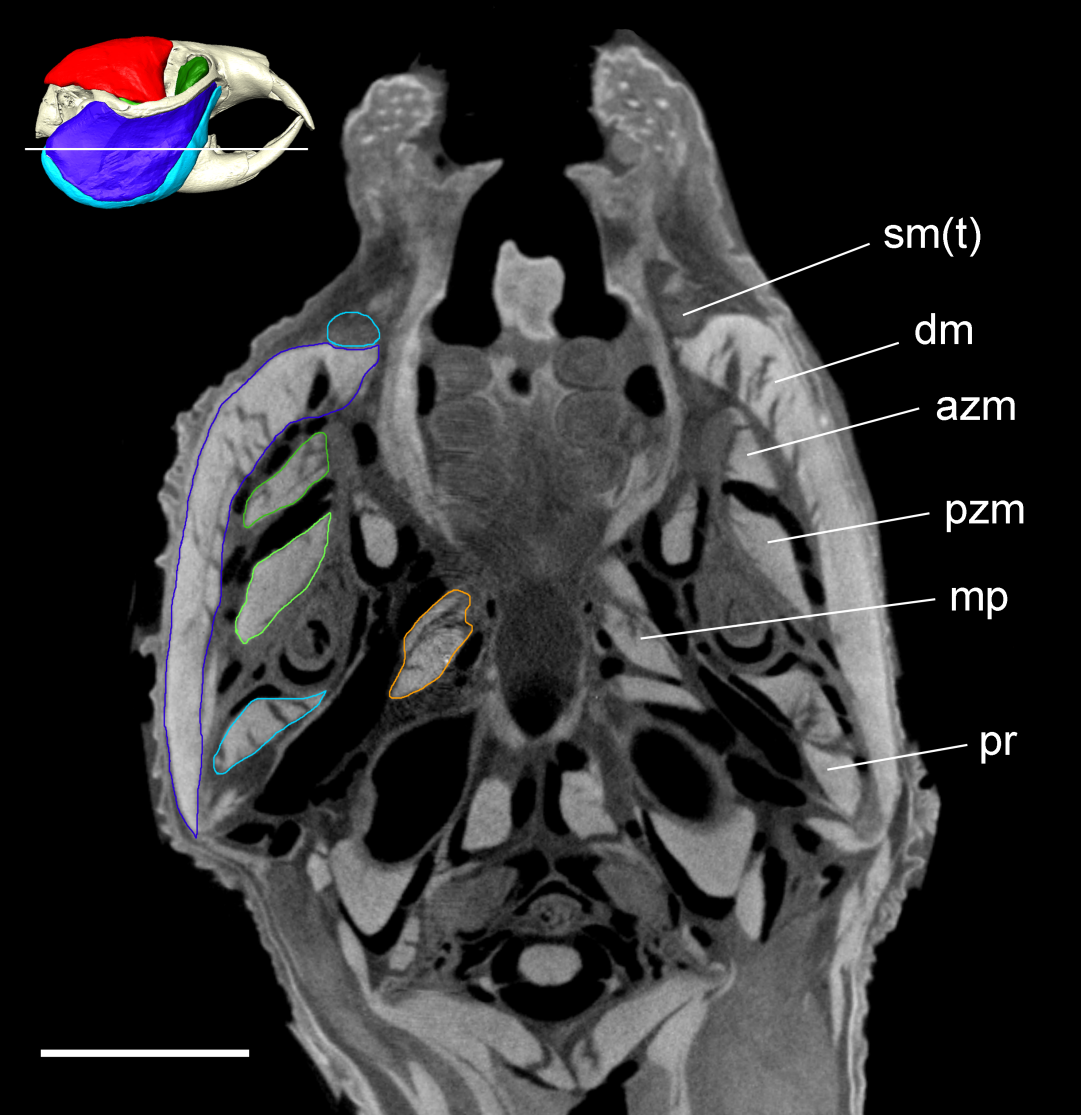
Transverse microCT slice of *Heterocephalus glaber*. Transverse microCT slice through the head of *Heterocephalus glaber* stained with iodine potassium iodide. Abbreviations: azm, anterior zygomaticomandibularis (dark green); dm, deep masseter (dark blue); mp, medial pterygoid (orange); pr, *pars reflexa* of the superficial masseter (light blue); pzm, posterior zygomaticomandibularis (light green); sm(t), tendon of superficial masseter (light blue). White line on 3D reconstruction shows position of slice. Scale bar = 5 mm.

**Figure 5:**
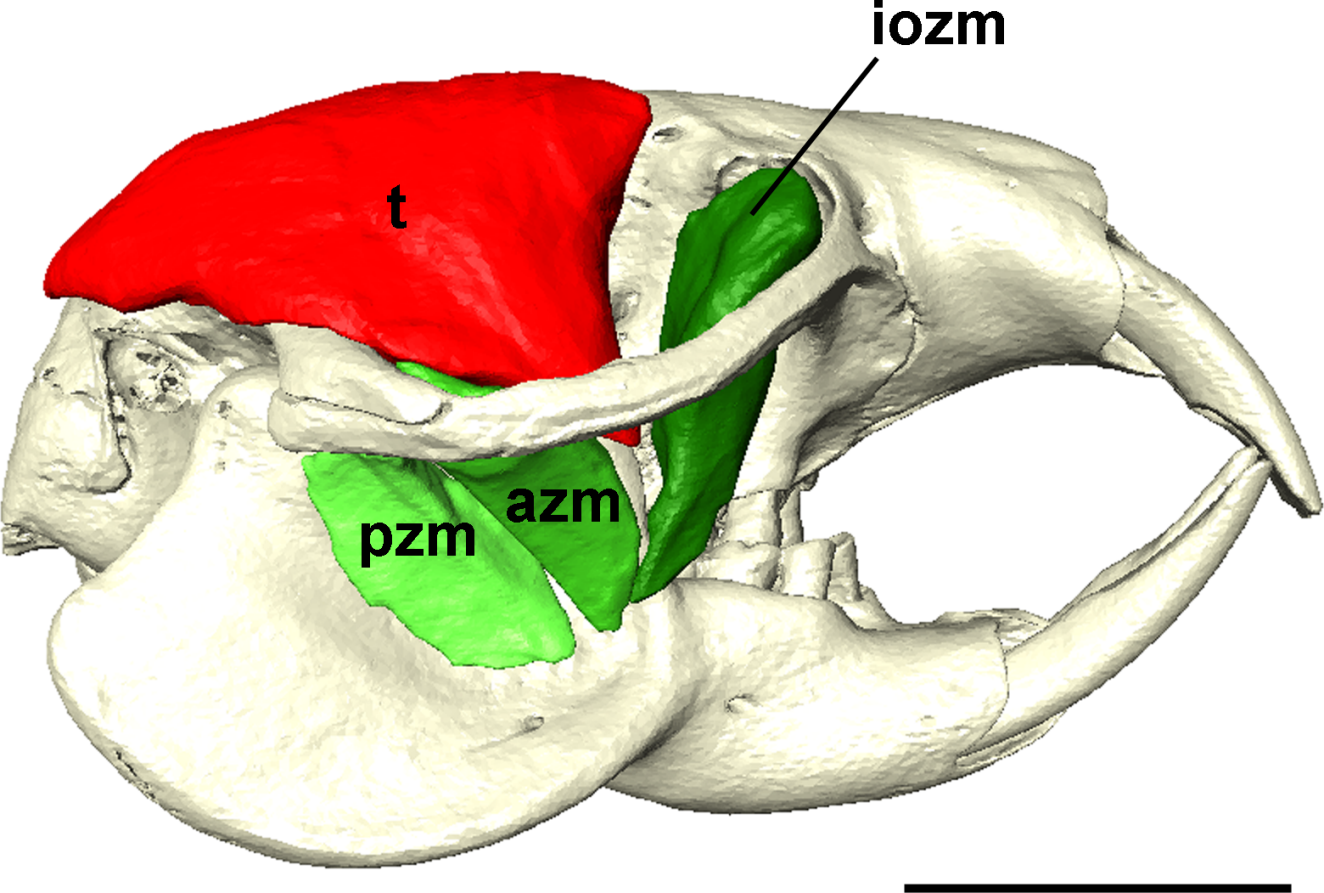
Temporalis and zygomaticomandibularis muscles of *Heterocephalus glaber*. Right lateral view of a 3D reconstruction of the cranium, mandible, temporalis and zygomaticomandibularis muscles of *Heterocephalus glaber*. Abbreviations: azm, anterior zygomaticomandibularis; iozm, infraorbital zygomaticomandibularis; pzm, posterior zygomaticomandibularis; *t*, temporalis. Scale bar = 5 mm.

The absolute masses and relative proportions of the jaw-closing muscles are given in Table 1. The muscles of mastication are described below and shown in Figs. 1–5. A 3D interactive reconstruction is provided in the supplementary PDF (File S1) and a rotating reconstruction is given in the supplementary movie (File S2).

**Table 1:** Masses and relative proportions of masticatory muscles of *Heterocephalus glaber*.

Muscle	Mass (g)	Percentage
Superficial masseter	0.057	23.4
Deep masseter	0.062	25.5
Anterior ZM	0.007	2.9
Posterior ZM	0.006	2.6
Infraorbital ZM	0.005	5.4
Temporalis	0.078	32.2
Medial pterygoid	0.015	6.1
Lateral pterygoid	0.013	2.0
Total	0.242	100.0

### Superficial masseter

The superficial masseter is a moderately large muscle in the naked mole-rat, forming just under a quarter of the masticatory muscle mass (Table 1). It takes a small tendinous origin from the ventral surface of the anterior zygomatic arch where it meets the skull. From its origin, the superficial masseter runs postero-ventrally along the anterior border of the deep masseter, and inserts along the ventral margin of the mandible. It also wraps around the mandibular margin and extends over the medial mandibular surface. This section of the superficial masseter, known as the *pars reflexa* (Turnbull, 1970), pushes backwards, around the insertion of the medial pterygoid, almost reaching the posteriormost tip of the medial surface of the angle (Figs. 2 and 3).

The microCT images of the naked mole-rat do not indicate that the superficial masseter is separated into smaller divisions. In most other descriptions of hystricomorph rodents, the superficial masseter is either divided into a *pars horizontalis* and a *pars reflexa* (e.g., Turnbull, 1970), or into a main section and a small *pars anterior* that originates from the mesial edge of the tendon (e.g., Woods, 1972; Woods & Hermanson, 1985; Hautier & Saksiri, 2009; Hautier, 2010). No *pars anterior* was identified in the naked mole-rat, but an extensive *pars reflexa* was clearly visible. Two previous descriptions of bathyergid musculature have reported a superficial masseter morphology not found in any other rodent species. Boller (1970) described a large superficial masseter in *Cryptomys* that completely covered the deep masseter in lateral view. He divided this muscle into three parts, termed M2, M1a and M1b, the latter being essentially synonymous with the superficial masseter described here. This configuration was also reported for *Fukomys* by Van Daele, Herrel & Adriaens (2009), although the authors note that the separation between the superficial masseter and deep masseter is very difficult. In this study, no part M1a or M2 could be identified, with all musculature dorsal to the superficial masseter being assigned to the deep masseter — a view shared by Morlok (1983).

### Deep masseter

The deep masseter is very similar in size to the superficial, forming 25% of the total muscle mass (Table 1). It lies immediately dorsal to the superficial masseter, originating along the length of the ventral border of the zygomatic arch and inserting on the masseteric ridge on the ventral part of the mandibular ramus (Fig. 4). As mentioned above, the deep masseter is not covered by part M2 of the superficial masseter as described by Boller (1970) and Van Daele, Herrel & Adriaens (2009), but is clearly visible in lateral view (as noted by Morlok, 1983). Thus, the naked mole-rat has a morphology similar to that seen in many hystricomorph taxa, such as *Proechimys* (Woods, 1972), *Myocastor* (Woods & Howland, 1979) and *Plagiodontia* (Woods & Hermanson, 1985). In some rodents, particularly sciuromorphs and myomorphs, the deep masseter has often been split into anterior and posterior portions (e.g., Greene, 1935; Weijs, 1973; Ball & Roth, 1995; Thorington & Darrow, 1996; Cox & Jeffery, 2011), but, in common with many descriptions of hystricomorphous rodents (e.g., Müller, 1933; Offermans & De Vree, 1993; Woods, 1972; Woods & Hermanson, 1985), no division of the deep masseter was found in *Heterocephalus glaber*.

A further division of the deep masseter, known simply as the ‘posterior masseter’ has been described in many hystricognaths (Woods, 1972; Woods & Howland, 1979; Woods & Hermanson, 1985; Hautier, Lebrun & Cox, 2012) and *Pedetes capensis* (Offermans & De Vree, 1989). The muscle originates in the jugal fossa on the ventrolateral surface of the zygomatic arch and runs horizontally and posteriorly to the condylar process of the mandible. Despite careful analysis of the microCT scans, no such muscle could be discerned in *Heterocephalus*, or has it been described in other bathyergids (Tullberg, 1899; Boller, 1970; Morlok, 1983; Van Daele, Herrel & Adriaens, 2009).

### Zygomaticomandibularis

The ZM is the deepest of the three muscle layers running from the zygomatic arch to the mandible. In the naked mole-rat, as in *Fukomys* (Van Daele, Herrel & Adriaens, 2009), it is clearly separable from the deep masseter that lies immediately lateral to it. The iodine-enhanced microCT images in this study show three distinct portions of the ZM (Fig. 5): (1) a posterior ZM originating from the glenoid fossa and running antero-ventrally to insert on the middle of the lateral mandibular ramus; (2) an anterior ZM with an origin spanning the jugo-squamosal suture on the medial surface of the zygomatic arch with an insertion on the ventral part of the coronoid process; and (3) an infraorbital ZM that originates from the anteriormost part of the orbit where the zygomatic arch meets the skull and the small infraorbital foramen pierces the maxilla and inserts at the base of the coronoid process lateral to the distal molar. No part of the infraorbital ZM was seen to pass through the infraorbital foramen, but this muscle was so named as it appears to be homologous with the infraorbital ZM in other rodents (e.g., Weijs, 1973; Olivares, Verzi & Vassallo, 2004; Satoh & Iwaku, 2006; Satoh & Iwaku, 2009; Cox & Jeffery, 2011).

Our description of the ZM largely matches that of Tullberg (1899) and Morlok (1983), differing only in nomenclature. Tullberg (1899) clearly identifies three portions of the medial masseter (equivalent to the ZM here), but assigns the rostral two portions to the ‘*Portio anterior masseteris medialis*’ rather than splitting them into infraorbital and anterior sections. Morlok (1983) identifies the three parts as (caudal to rostral): (1) posterior ZM, (2) anterior ZM, and (3) ‘*maxillo-mandibularis*’. There are, however, substantial differences in the muscle arrangement described by Boller (1970) in *Cryptomys*. The middle part, here identified as the anterior ZM, was determined by Boller (1970) to be a ventral extension of the temporalis muscle (*pars zygomatica*) on to the lateral surface of the mandible. This was not thought to be the case in the naked mole-rat specimen scanned for this study as, in common with Morlok (1983) no clear connection between this muscle and the temporalis could be seen.

### Temporalis

The temporalis is the largest jaw-closing muscle in *Heterocephalus glaber*, forming around 32% of the masticatory muscle mass (Table 1). Its origin covers the entire parietal bone and much of the frontal and squamosal as well, extending from the dorsal midline down to the zygomatic process of the squamosal, and from the occipital bone across the braincase well into the orbit (Fig. 5). The insertion is on the tip and widely across the medial surface of the coronoid process.

### Medial pterygoid

The medial pterygoid is fairly small component of the masticatory complex in the naked mole-rat, comprising just 6% of the total jaw-closing muscle mass (Table 1). It is split into two branches at its origin — a small branch that attaches to the lateral surface of the pterygoid plate, and a much larger branch that originates deep within the pterygoid fossa. The fossa opens into the braincase in *Heterocephalus glaber* as it does in all bathyergids and all hystricognaths except *Hydrochoerus* (Woods, 1972). The two branches of the medial pterygoid unite and run caudally, ventrally and laterally to take a long, narrow insertion on the medial surface of the angle of the mandible (Fig. 3). The insertion of the medial pterygoid is almost completely surrounded by the *pars reflexa* of the superficial masseter (Figs. 2 and 3).

### Lateral pterygoid

The lateral pterygoid originates from the lateral pterygoid plate and part of the alisphenoid bone, dorsal to the smaller branch of the medial pterygoid. From its origin it extends postero-laterally, in an almost horizontal plane, to the medial surface of the mandibular condyle (Fig. 3). The insertion is immediately dorsal to the *pars reflexa* of the superficial masseter. The muscle is very small, forming around 2% of the masticatory musculature (Table 1).

## Discussion

The technique of iodine-enhanced microCT scanning (Metscher, 2009; Jeffery et al., 2011) was used to visualise the jaw-closing musculature of the naked mole-rat. Although the specimen studied had been preserved for several years in ethanol and undergone a substantial amount of muscle shrinkage, the iodine potassium iodide staining was very successful in revealing the different layers and sections of the masticatory muscles.

The results show that the masticatory muscles of the naked mole-rat are very large. Although the absolute muscle masses are of limited use, given the extensive muscle shrinkage that has taken place, it should be noted that the total muscle masticatory mass (0.242 g) is 75% of that reported for the rat (Cox & Jeffery, 2011), despite the naked mole-rat skull being under half the length of the rat skull, and the average body mass of non-breeding adult naked mole-rats in captivity (30–50 g; Jarvis & Sherman, 2002) being between 14 and 23% of the body mass estimated for the rat specimen used by Cox & Jeffery (2011). Thus, even before muscle shrinkage has been accounted for, the naked mole-rat clearly has very large jaw-closing muscles compared to other rodents. In fact, Jarvis & Sherman (2002) report that the jaw muscles constitute around a quarter of the entire muscle mass of the naked mole-rat.

The masticatory complex is dominated by three muscles: the superficial masseter, the deep masseter and the temporalis. The superficial and deep masseters together form almost 50% of the masticatory musculature. Gorniak (1977) and Byrd (1981) suggested that these muscles have an important role in the closing and power strokes of biting at both the incisors and molars. Thus, the large masseter in the naked mole-rat is likely to deliver a high bite force. In addition, the strong horizontal component of pull in the superficial masseter is likely to make this muscle the main protractor of the lower jaw, as proposed by Hiiemae (1971). The microCT images revealed a large portion of the superficial masseter that wraps around the ventral margin of the mandible and inserts on the medial surface of the ramus, known as the *pars reflexa*. Various functions have been suggested for this part of the muscle, including fine control of jaw opening (Weijs & Dantuma, 1975) and increase in the resting length of the muscle to facilitate wider gapes (Satoh & Iwaku, 2004). The lack of posterior masseter in the naked mole-rat is somewhat unusual, given its presence in most other hystricognaths (Woods, 1972; Woods & Howland, 1979; Woods & Hermanson, 1985). However, it is perhaps not surprising as its presence seems to be correlated with hystricomorphy (a posterior master is found in *Pedetes* as well; Offermans & De Vree, 1989) and it has not been reported in other bathyergids (Tullberg, 1899; Boller, 1970; Morlok, 1983; Van Daele, Herrel & Adriaens, 2009). Offermans & De Vree (1993) found that the function of the posterior masseter was largely propalineal movement of the mandible, so it may be that such motion of the jaws is not important for mole-rats. Indeed, observations indicate that jaw movements are in fact mainly oblique in bathyergids (H Gomes Rodrigues, pers. comm., 2014).

The temporalis muscle in the naked mole-rat extends across the entire temporal region of the cranium and also pushes into the orbit. As a proportion of total jaw adductor muscle mass (32%), it is very large compared to other species in the Hystricognathi e.g., *Cavia* (11%: Cox & Jeffery, 2011), *Hydrochoerus* (5%: Müller, 1933) and *Hystrix* (17%: Turnbull, 1970). However, it should be noted that temporalis muscles of similar relative size have been recorded in *Aplodontia rufa* (34%) and many sciuromorphs (25%–30%) by Druzinsky (2010), and may in fact be the primitive condition for rodents. The highly reduced size of the eye in the naked mole-rat may have partly facilitated the increase in size of the temporalis, enabling its anterior expansion into the relatively unoccupied orbit (Lavocat, 1973). It has also been suggested that a large temporalis may result from the widening and flattening of the skull seen in fossorial species (Druzinsky, 2010). Such a large temporalis is likely to be acting as a powerful elevator of the jaw (Hiiemae, 1971), producing substantial forces at the teeth. The most ventral fibres of temporalis that run along the zygomatic process of the squamosal have a largely horizontal direction of pull, and thus are likely to act as a strong retractor of the jaw. Overall, it appears that the two largest masticatory muscles of the naked mole-rat may provide it with the potential to generate high bite forces and to produce a wide gape. Both these are highly useful characteristics in a subterranean species that digs extensive tunnels in search of widely dispersed food resources with its incisors, as the naked mole-rat does (Stein, 2000). Brett (1991) radio tracked individuals in a colony of 87 naked mole-rats and found that 3.6–4.5 tonnes of soil were excavated in a single year — the equivalent of 2.3–2.9 km of new tunnels. Such endeavours impose high costs with respect to tooth wear, yet incisor growth is not dissimilar to that of other rodents (Berkovitz & Faulkes, 2001), and perhaps offset as a limiting factor by the social behaviour of the naked mole-rat, where digging activity is distributed among a large workforce with strong jaws. It is most likely that digging through hard soil is the main constraint on jaw anatomy and musculature, as the underground roots and tubers on which naked mole-rats feed are not woody or particularly tough to chew. For example, one of the most common geophytes eaten by naked mole-rats in Kenya (*Pyrenacantha*) has a succulent flesh with just a thin epidermis (<1mm thick), and this is typical of the other food plant species they consume (Brett, 1991).

The iodine-enhanced microCT scans show very clearly that the zygomaticomandibularis muscle does not pass through the infraorbital foramen and on to the rostrum in *Heterocephalus glaber*. Thus, the naked mole-rat displays the protrogomorphous morphology (Wood, 1965), as do most other genera in the Bathyergidae (Tullberg, 1899), although there is a very small extension of the ZM through the infraorbital foramen in *Cryptomys* (Boller, 1970; Morlok, 1983) and *Fukomys* (Van Daele, Herrel & Adriaens, 2009). In contrast, all other families in the Hystricognathi are hystricomorph (i.e., they possess a large infraorbital portion of the ZM that passes through an enlarged infraorbital foramen and takes a wide origin on the rostrum). However, the 3D reconstructions demonstrate that, although the naked mole-rat is technically protrogomorphous, it is very different in morphology to the other extant protrogomorph, *Aplodontia rufa*. In the mountain beaver, the origin of the ZM is restricted to the medial surface of the zygomatic arch and the internal surface of the maxillary root of the zygoma (Druzinsky, 2010). It does not have the wide attachment in the anterior orbit seen in *Heterocephalus glaber*. The morphology of the ZM in the naked mole-rat very much resembles the hystricomorphous condition without the extension on to the rostrum. This morphology concurs with the assumption of secondarily lost hystricomorphy, based on the position of the Bathyergidae within the rodent phylogeny (Fabre et al., 2012), and the presence of hystricomorphy in fossil genera (Lavocat, 1973) and ontogeny (Maier & Schrenk, 1987). It appears that most of the blesmols, *Heterocephalus* included, have undergone a shortening of the rostrum (Landry, 1957), which may account for the retreat of the ZM from the snout. Such a rostral shortening is also seen in other fossorial hystricognaths e.g., *Ctenomys* (Vassallo & Verzi, 2001) which also has a relatively reduced infraorbital part of the ZM compared to the semi-fossorial *Octodon* and the terrestrial *Chinchilla* (Becerra et al., 2014). It should be noted that shortening of the rostrum would shorten the out-lever of the masticatory system, thus contributing to high bite forces. The loss of the infraorbital ZM may also have been an adaptation (alongside the enlarged *pars reflexa* of the superficial masseter, mentioned above) to increasing gape for incisor digging. Overall, the condition of the muscles in *Heterocephalus* is also a reminder that rodent masticatory muscles are in fact a suite of continuous characters, and that imposing discrete descriptive terms (protrogomorph, hystricomorph) on them may result in erroneous interpretations of evolutionary history and relationships.

In conclusion, the naked mole-rat has evolved an enlarged set of masticatory muscles, particularly the superficial masseter and temporalis. These large muscles may contribute, alongside rostral shortening, to a presumably high bite force and wide gape necessary for digging with the incisor teeth. The overall morphology is protrogomorphous, but is consistent with evolution from a hystricomorphous ancestor, with the infraorbital portion of the zygomaticomandibularis having been lost possibly through rostral shortening. The contrast-enhanced microCT technique has been shown to be a highly effective tool for the visualisation of soft tissues, especially muscle. Although a great deal of tissue shrinkage is evident in the scans presented here, this is mostly an artifact of the lengthy fixation time; such extreme shrinkage is highly unlikely in fresher specimens. It is hoped that iodine-enhanced microCT will become part of the standard toolkit of anatomical investigation in the future.

## Supplemental Information

10.7717/peerj.448/supp-1 File S1 Digital dissection of the masticatory musculature of *Heterocephalus glaber* Interactive 3D PDF showing cranium, mandible and masticatory musculature of *Heterocephalus glaber*. Abbreviations: ZM, zygomaticomandibularis.

10.7717/peerj.448/supp-2 File S2 Masticatory musculature of *Heterocephalus glaber* Virtual reconstruction of the cranium, mandible and masticatory musculature of *Heterocephalus glaber* rotated through 360°.

## Additional Information and Declarations

### Data Deposition

The following information was supplied regarding the deposition of related data: The data is deposited in Hydra, the University of Hull data repository under a creative commons BY-NC-SA licence. The accession number is 8475. Link: https://hydra.hull.ac.uk/resources/hull:8475
