# Supplementary figures and images for: Digital dissection of the masticatory muscles of the naked mole-rat, Heterocephalus glaber (Mammalia, Rodentia)
Source: PeerJ. 2014 Jun 17;2:e448. doi: 10.7717/peerj.448 (PMC4081180; doi:10.7717/peerj.448)

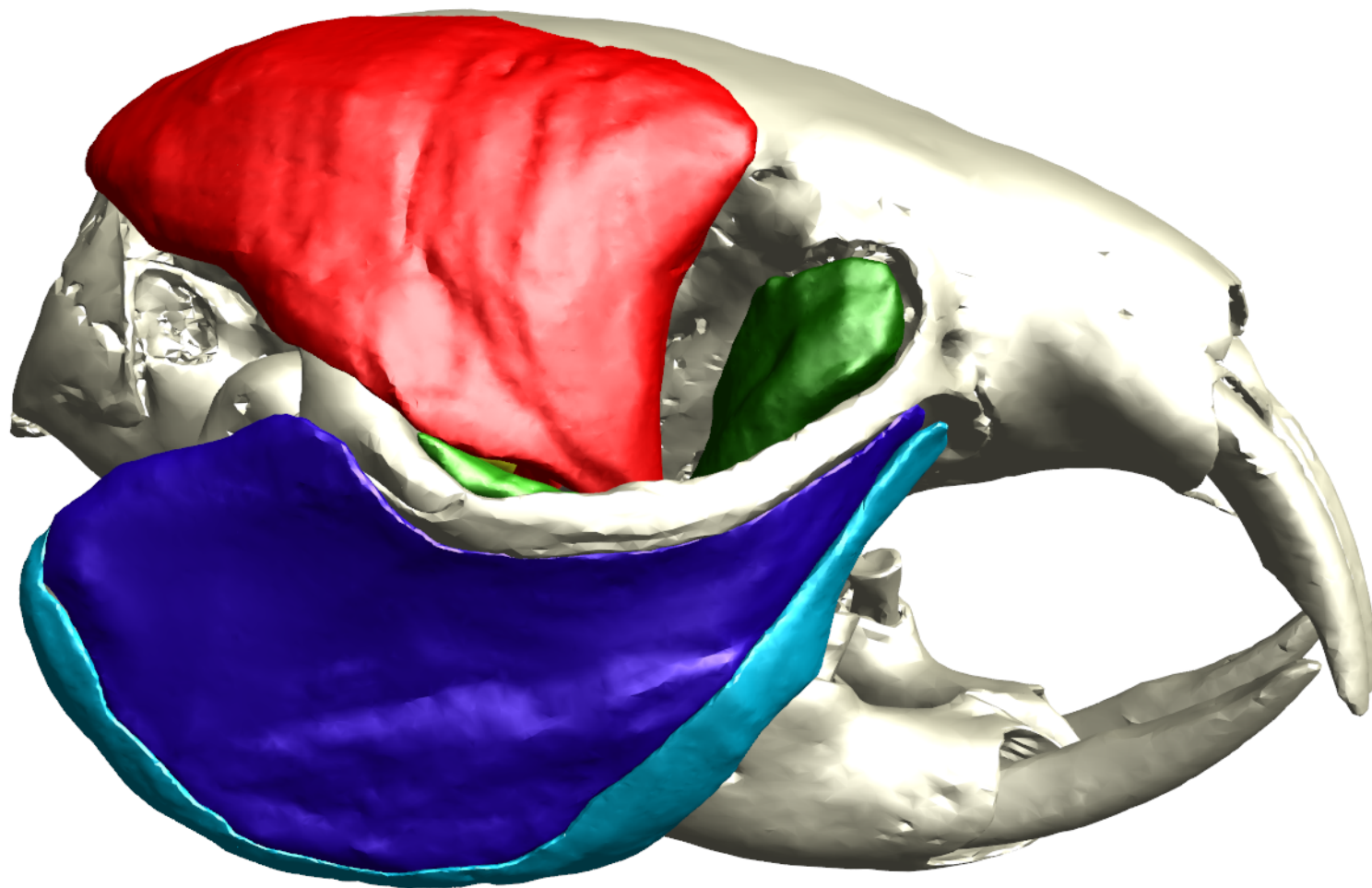

Supplement: File S1 — Interactive 3D PDF showing cranium, mandible and masticatory musculature of Heterocephalus glaber. Abbreviations: ZM, zygomaticomandibularis. [file peerj-02-448-s001.pdf]
